# Different effects of tryptophan 2,3-dioxygenase inhibition on SK-Mel-28 and HCT-8 cancer cell lines

**DOI:** 10.1007/s00432-020-03351-2

**Published:** 2020-08-10

**Authors:** Sara Paccosi, Marta Cecchi, Angela Silvano, Sergio Fabbri, Astrid Parenti

**Affiliations:** 1 Department of Health Sciences, Clinical Pharmacology and Oncology Section, University of Florence, Viale Pieraccini 6-50139, Florence, Italy; 2 Department of Biomedical, Experimental and Clinical Sciences, University of Florence, Viale Pieraccini 6, 50139 Florence, Italy

**Keywords:** Tryptophan 2,3-dioxygenase, 680C91, Melanoma, SK-mel-28, HCT-8, Trp metabolism

## Abstract

**Purpose:**

Indoleamine 2,3-dioxygenase-1 (IDO1) and more recently, tryptophan 2,3-dioxygenase (TDO), are tryptophan-catabolizing enzymes with immunoregulatory properties in cancer. IDO1 is more expressed than TDO in many tumours including melanomas; however, IDO inhibitors did not give expected results in clinical trials, highlighting the need to consider TDO. We aimed to characterize both TDO expression and function in a melanoma cell line, named SK-Mel-28, with the purpose to compare it with a colon cancer cell line, HCT-8, and with a human endothelial cell line (HUVEC).

**Methods:**

TDO expression was assessed as real time-PCR and western blot, for mRNA and protein expression, respectively. While cell proliferation was assessed as cell duplication, cell apoptosis and cell cycle were analysed by means of flow cytometry.

**Results:**

SK-Mel-28 cells showed higher TDO levels compared to HCT-8 and to HUVEC cells. A selective TDO inhibitor, 680C91, significantly impaired cell proliferation in a concentration-dependent manner, by inducing cell arrest during the G2 phase for SK-Mel-28 and HUVEC cells, while an early apoptosis was increasing in HCT-8 cells. No toxic effects were observed. These data demonstrated that TDO is highly expressed in SK-Mel-28 cells and may be involved in the regulation of their proliferation.

**Conclusion:**

TDO may directly modulate cancer cell function rather than immune suppression and can be considered as a target for melanoma progression together with IDO1.

## Introduction

Tryptophan (trp) degradation is a mechanism employed by a broad range of tumours to suppress the immune response. Two distinct enzymes are involved in its catabolism: indoleamine 2,3-dioxygenase (IDO1 and 2) and tryptophan 2,3-dioxygenase (TDO) that catalyse the first and rate limiting step of trp oxidation yielding kynurenine (kyn). The kynurenine pathway (KP) produces many biologically active metabolites, including the redox oxidized cofactors nicotinamide adenine dinucleotide (phosphate) [NAD + (P +)] and its reduced form NAD(P)H. Many data are reported on IDO1 and immune escape. Overexpression of IDO1 gene contributes to the depletion of local trp and to the elevation of kyn in the tumour microenvironment that makes it immunosuppressive. T-cell and NK-cell are indeed sensitive to kyn accumulation that inhibits NK cell function, favours the differentiation of regulatory T lymphocytes (T reg) and suppresses proliferation and activity of effector T lymphocytes (Terness et al. 2002; Uyttenhove et al. 2003; Opitz et al. 2011). The sequence similarity between human IDO1 and TDO is low (16%), however maintaining a high similarity in their catalytic domains (Thackray et al. 2008). While TDO is specific for metabolizing tryptophan to kyn, IDO1 recognizes a broad range of indole-containing substrates including the neurotransmitter melatonin. Moreover, they have different tissue distributions and physiological roles; IDO is a monomeric enzyme less expressed in normal tissues and upregulated during inflammation, to suppress immune reaction; TDO is a tetramer and it is highly expressed in the liver where it degrades excesses of dietary trp (Yu et al. 2017), in the placenta where it regulates the immune response (Yu et al. 2017), and in the brain where it regulates the synthesis of neurotransmitters (Beatty and Gladney 2015). TDO expression and its role within cancer has been poorly investigated until a few years ago, while the proven role of IDO in cancer biology has resulted in its extensive study and in the identification of pharmacological inhibitors. IDO1 is indeed upregulated in many solid tumours, contributes to immunologic evasion and it is associated with poor patient outcomes (Pilotte et al. 2012; Yu et al. 2018). Conversely, TDO2 mRNA expression has been found in some tumour cell lines and also in human tumours, such as hepatocarcinoma, melanoma and glioblastoma (van Baren and Van den Eynde 2015), cutaneous melanoma (Pilotte et al. 2012) and primary uveal melanoma, in which mRNA expression is generally low but its increase is associated with poor prognostic markers (Terai et al. 2020). TDO-mediated immunosuppression has been reported to be mediated by the human aryl hydrocarbon receptor (Ahr) (Platten et al. 2012). Its activation induces the activation of the Foxp3 in naïve T lymphocytes preventing the maturation of Th17 lymphocytes and a lower cell-mediated response, through the antigen presenting dendritic cells (DC), stimulating the selective proliferation of Treg and therefore promoting an immunosuppressive environment (Platten et al. 2012). In addition, it has been demonstrated that in Ahr-proficient mice the expression of TDO strongly enhanced tumour growth in comparison with tumours not expressing TDO (Opitz et al. 2011).

The importance of trp/kyn homeostasis in immune escape has made IDO1 and TDO good targets for the development of inhibitors. Despite promising results of some IDO1 inhibitors alone or in combination with PD1 inhibitors, their benefits in melanoma patients have not been completely demonstrated (Long et al. 2019). The expression and function of TDO in melanoma is not clear yet, and a further investigation is necessary.

Based on these considerations, we aimed to extend the knowledge of TDO expression and function. We evaluated the expression and the possible function of TDO in a human melanoma cell line, SK-Mel-28, in which TDO has never been characterized up to now, in a human colorectal cancer cell line HCT-8 and in human endothelial cells HUVEC.

## Methods

### Cell culture

A human metastatic melanoma cell line, SK-Mel-28 (ATCC, Manassas, VA), a human colon adenocarcinoma cell line, HCT8, and HUVEC cells (ECACC, Salisbury UK) were grown on high D-glucose DMEM, RPMI and on M199, respectively. Media were supplemented with 10% (v/v) heat inactivated fetal bovine serum (FBS Defined Hyclone; Thermo Scientific, Waltman, MA), 100 U/mL penicillin, 100 μg/mL streptomycin and 2 mmol/L glutamine in a humidified atmosphere with 5% CO_2_ in air. The culture medium utilized, a “complete medium”, was changed every 2 days.

### Real Time PCR

The total RNA was isolated from SK-Mel-28, HCT8 and HUVEC cells, using TRI Reagent and quantified spectroscopically with a NanoDrop (Thermo-fisher scientific, Waltham, Massachusetts, USA). One μg of RNA was used for the reverse transcription reaction with Prime Script RT reagent Kit with gDNA eraser (Takara, Otsu, Japan) and the cDNA samples obtained were amplified with specific primers described below. TDO2 amplification fw: 5′-CTTATCTCCAGCATCAGGCTTCCAGAGT-3′ and rev: 5′-GGAGTTCTTTCCAGCCATGCCTCC-3′.

Real-time PCR assays were carried out using SYBR Premix Ex Taq (Takara, Otsu, Japan) according to the manufacturer instructions on a Rotorgene RG-3000A cycle system (Qiagen, Germany) platform. PCR amplification of 18 s ribosomal mRNA was used as a normalizer. 18 s amplification fw: ATTAAGGGTGTGGGCCGAAG and rev: GGTGATCACACGTTCCACCT.

The cycle was set at 95 °C for 5 s, followed by 55 °C for 30 s, repeated 35 times. Quantitative real-time polymerase chain reaction data analysis was accomplished with delta delta CT method.

### Cell proliferation

Cell proliferation was quantified by total DNA/well via a fluorescent dye (cell proliferation kit, Invitrogen) (Paccosi et al. 2012). Briefly, cells (1.5 × 10^3^/100 μl) were plated on flat-bottom 96-multiwell plates and allowed to adhere overnight. Cells were kept in starving conditions (1% FCS) for 24 h in the presence or absence of 680C91 (5–40 μM), then media were removed and replaced with 10% FCS medium. After 48 h, 100 μl of dye binding solution were added to each microplate well and incubated at 37 °C for 30 min. Fluorescence intensity was read using a fluorescence microplate reader with excitation at ~ 485 nm and emission detection at ~ 530 nm.

### Cell cycle

Cell cycle analysis was performed by means of flow cytometry. Cells were starved for 24 h in 1% FCS in the presence or absence of 680C91 (20–40 μM). Then, they were harvested, washed in PBS and centrifuged at 280 g. Cellular pellets were fixed with 200 µl of 70% cold ethanol and incubated for 30 min in ice. After two washes and centrifuge at 850 g for 7 min, the pellet containing nucleic acid was incubated with 50 µl of a ribonuclease-A, 100 µg/ml solution, to digest RNA. After 30 min, cellular DNA was incubated with propidium iodide (PI) at a final concentration of 50 µg/ml. Cell cycle was measured with FACSCanto II™ flow cytometer (BD Biosciences, San Jose, CA) emitting at 488 nm, which provided PI excitation in the blue-to-green range which is related to DNA content in each phase of cell cycle. Histograms of that emission were evaluated on linear scale with FCS express 6.0 software (De Novo Software, Pasadena, CA).

### Cell apoptosis

Apoptosis was assessed by flow cytometry as previously reported (Grassia et al. 2010). Briefly, cells were starved in 1% FCS for 24 h in the presence or absence of 680C91 (10–40 μM), then harvested and stained with FITC-annexin V and propidium iodide (PI) in PBS with Ca^2+^ and Mg^2+^ for 15 min in dark. Stained cells were immediately subjected to flow cytometry analyses using FACS Canto II flow cytometer (BD Biosciences). Positive control was treated with 500 μM cumene hyroperoxide (CHPx) for 3 h before staining.

### Western blot analysis

SK-Mel-28, HCT8 and HUVEC cells were lysed in a Triton^®^ X-100 lysis buffer, pH 7.4, followed by a centrifugation at 14,000 rpm for 10 min at 4 °C (Paccosi et al. 2012). Cell lysate was run on 10% SDS–polyacrylamide gel electrophoresis, blotted onto PVDF membrane (Merk-Millipore, Darmstadt, Germany) and immunostained with mouse monoclonal anti-TDO antibody (1:2000) and with anti β-tubulin monoclonal antibody (1:1000). The antigen–antibody complexes were visualized using appropriate secondary antibodies and the ECL detection system, as recommended by the manufacturer (Amersham Corp.).

### Material

680C91 ((E)-6-fluoro-3-[2-(3-pyridyl)vinyl]-1H-indole), ribonuclease-A, CHPx, anti β-tubulin monoclonal antibody, TRI Reagent were from Merck (KGaA, Darmstadt, Germany); Annexin V was from ImmunoTools GmbH (Gladiolenweg 2; Germany); mouse monoclonal anti-TDO antibody was from NovusBio (Bio-Techne SRL, MI, Italy); propidium iodide (PI) (BD Biosciences); high D-glucose DMEM, RPMI 1640, M199 and PBS were from Euroclone S.p.A. (MI, Italy).

### Statistical evaluation

Statistical analysis was performed with Prism software (GraphPad). Parametric data were reported as means ± SEM and differences between groups were tested with ANOVA test (followed by Bonferroni’s and Dunnett’s Multiple Comparison Test) as appropriate. Alpha value was set at 0.05.

## Results

### TDO expression

**Fig. 1:**
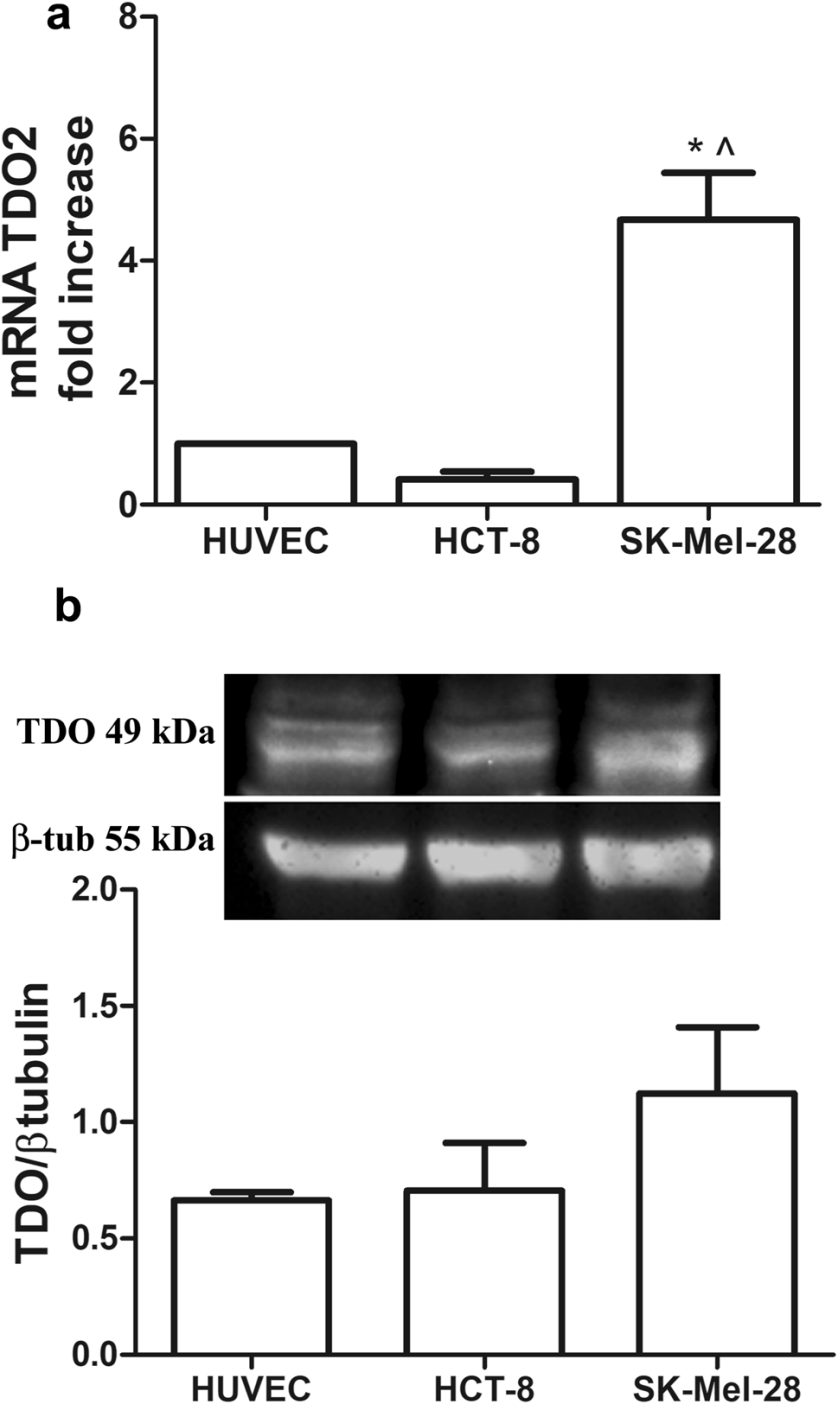
RT-PCR for TDO mRNA (TDO2, **a**) and protein (**b**) in HUVEC, HCT-8 and SK-Mel-28; Mean ± SEM, *n* = 4, *P < 0.001 vs HUVEC; ^*P* < 0.001 vs HCT-8

We investigated TDO expression as both mRNA (TDO2) and protein levels in SK-Mel-28, HCT-8 and HUVEC cells. TDO2 mRNA expression was significantly higher in SK-Mel-28 cells, compared to the human colon adenocarcinoma cell line and to the human endothelium, being 4.8 ± 0.9 fold over HUVEC and 12.5 ± 5 over HCT-8 (Fig. 1a). Western blot in reducing conditions confirmed protein expression of a monomer of 49 kDa (Fig. 1b).

### Effect of the selective TDO inhibitor 680C91 on cell proliferation

**Fig. 2:**
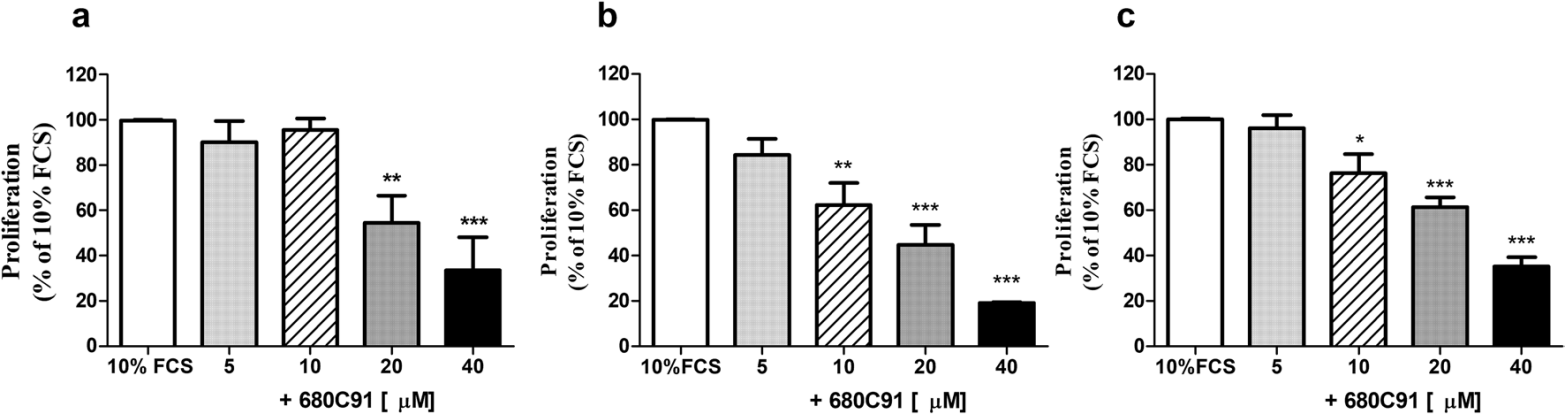
Effect of the TDO inhibitor 680C31 on SK-Mel-28 (**a**) HCT-8 (**b**) and HUVEC proliferation (**c**). Mean ± SEM, *n* = 6; *P < 0.05 **P < 0.01, ***P < 0.001 vs 10% FCS

The effect of 680C91, a selective TDO inhibitor, on SK-Mel-28, HCT8 and HUVEC cells growth was assessed. The addition of 680C91 (5–40 μM), significantly impaired cell proliferation in a concentration-dependent manner (Fig. 2b, c), whose maximal effect was observed at 40 μM, concentration capable to inhibit cell growth by 97.1 ± 2.3%, 81.5 ± 0.5% and 65 ± 3.7%, for SK-Mel-28, HCT8 and HUVEC, respectively. At the highest concentration, (40 μM the TDO inhibitor did not affect basal proliferation and it was free of any toxic effect (data not shown).

### Effect of the selective TDO inhibitor 680C91 on cell cycle

**Fig. 3:**
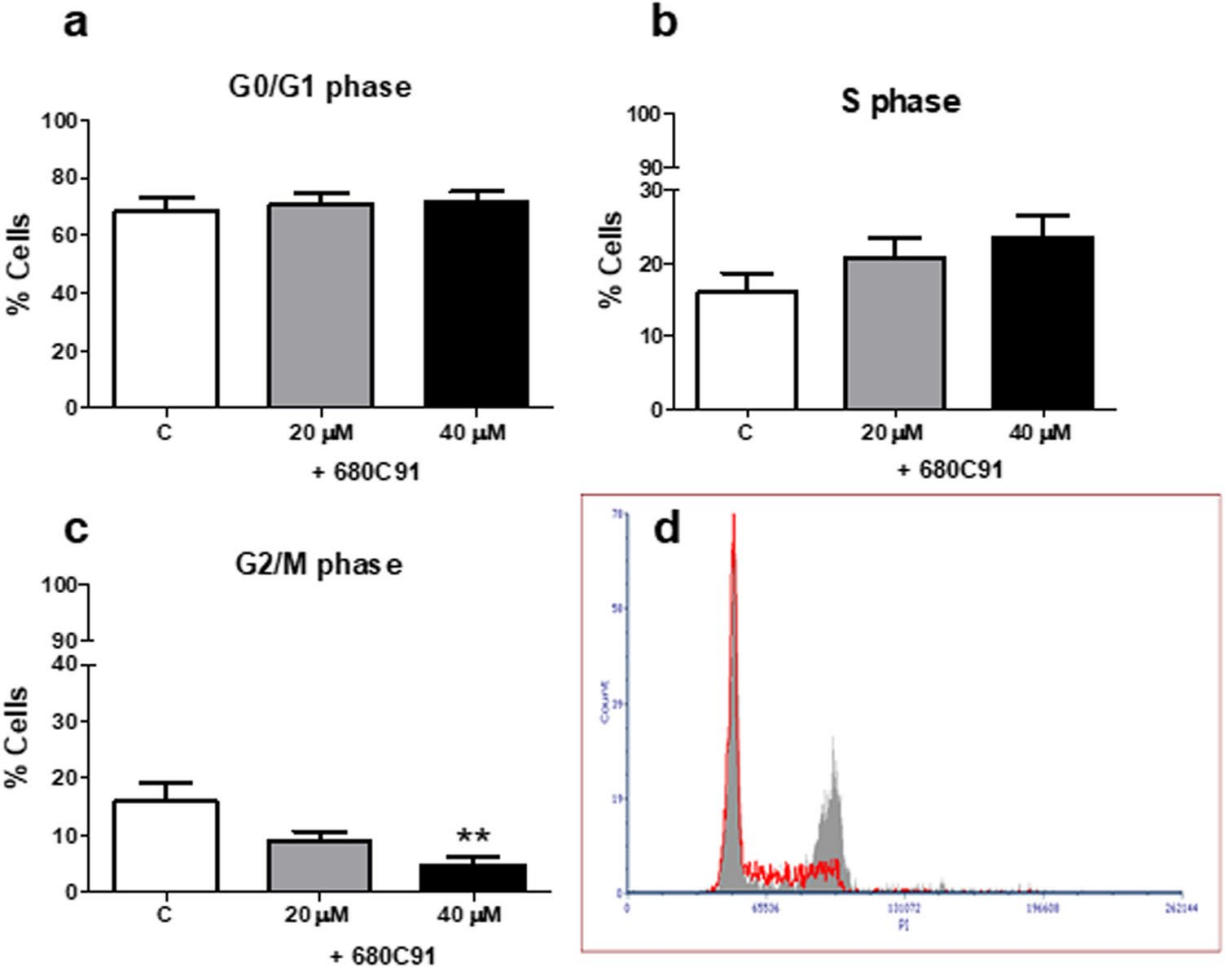
Effect of the TDO inhibitor 680C31 (20 and 40 µM) on SK-Mel-28 cell cycle. Mean ± SEM, *n* = 3; **P < 0.01, vs control unstimulated cells (C). Cell cycle distribution of propidium iodide (PI)-labelled cells was analysed by flow cytometric analyses. **a** G0/G1 phase; **b** S phase; **c** G2/M phase; **d** Close to bar graphs there is a representative histogram of control cells (grey line) and 680C91-treated cells (red line)

The analysis of the cell cycle phase distribution was performed to deepen the antiproliferative effects observed in presence of the TDO inhibitor. The cell cycle phase distribution was analysed following a 24 h-treatment with 680C91 at concentrations of 20 and 40 µM. Figure 3 shows that the percentage of SK-Mel-28 in the G2/M phase significantly decreased in a dose-dependent manner, compared to the unstimulated cells (the control cells), with a maximal effect at 40 µM (− 79%). The decrease in the G2/M phase was accompanied by no significant differences in the G0 and S phases.

**Fig. 4:**
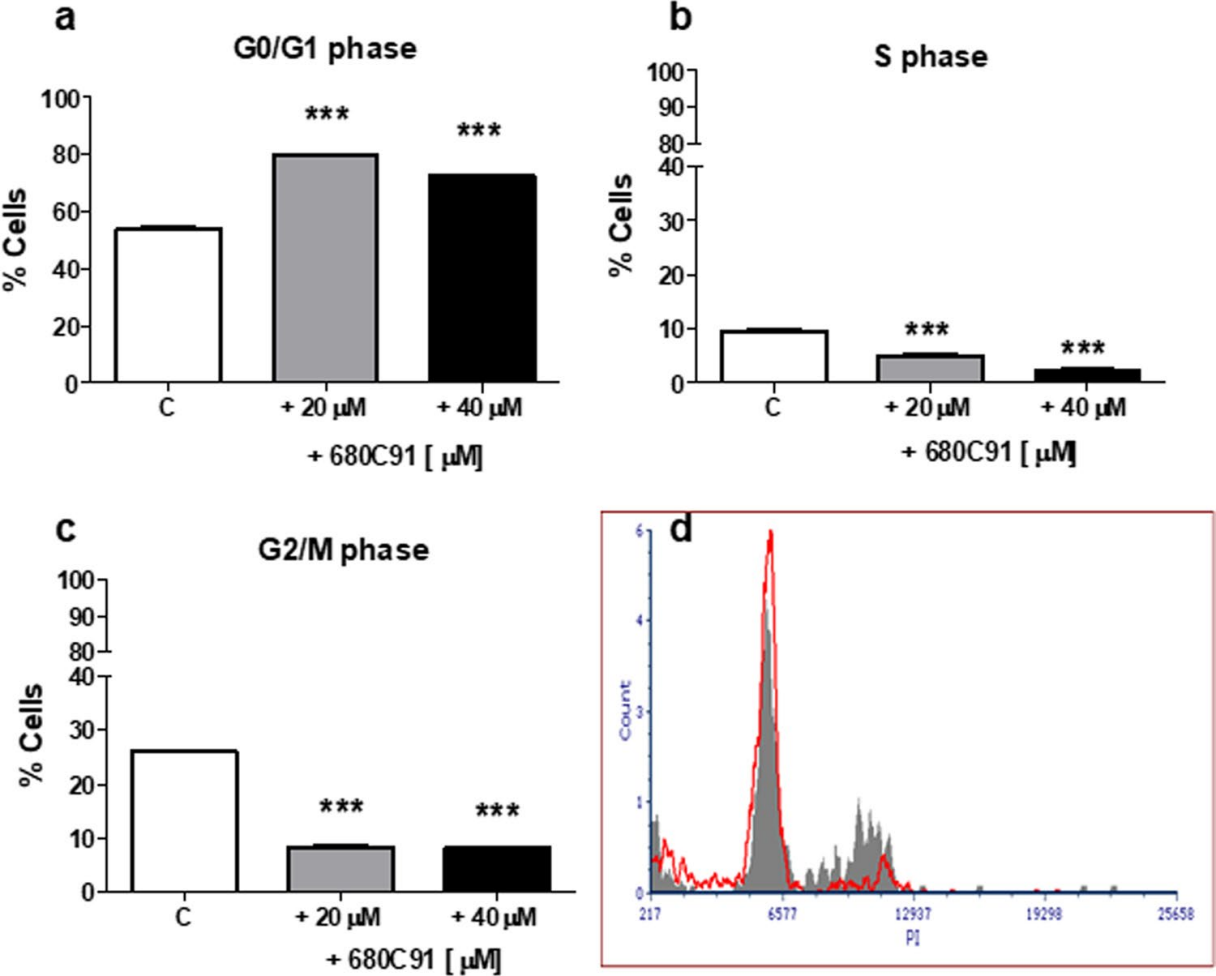
Effect of the TDO inhibitor 680C91 (20 and 40 µM) on HUVEC cell cycle. Mean ± SEM, *n* = 3 **P < 0.01, vs control unstimulated cells (C). **a** G0/G1 phase; **b** S phase; **c** G2/M phase; **d** close to bar graphs there is a representative histogram of control cells (grey line) and 680C91-treated cells (red line)

**Fig. 5:**
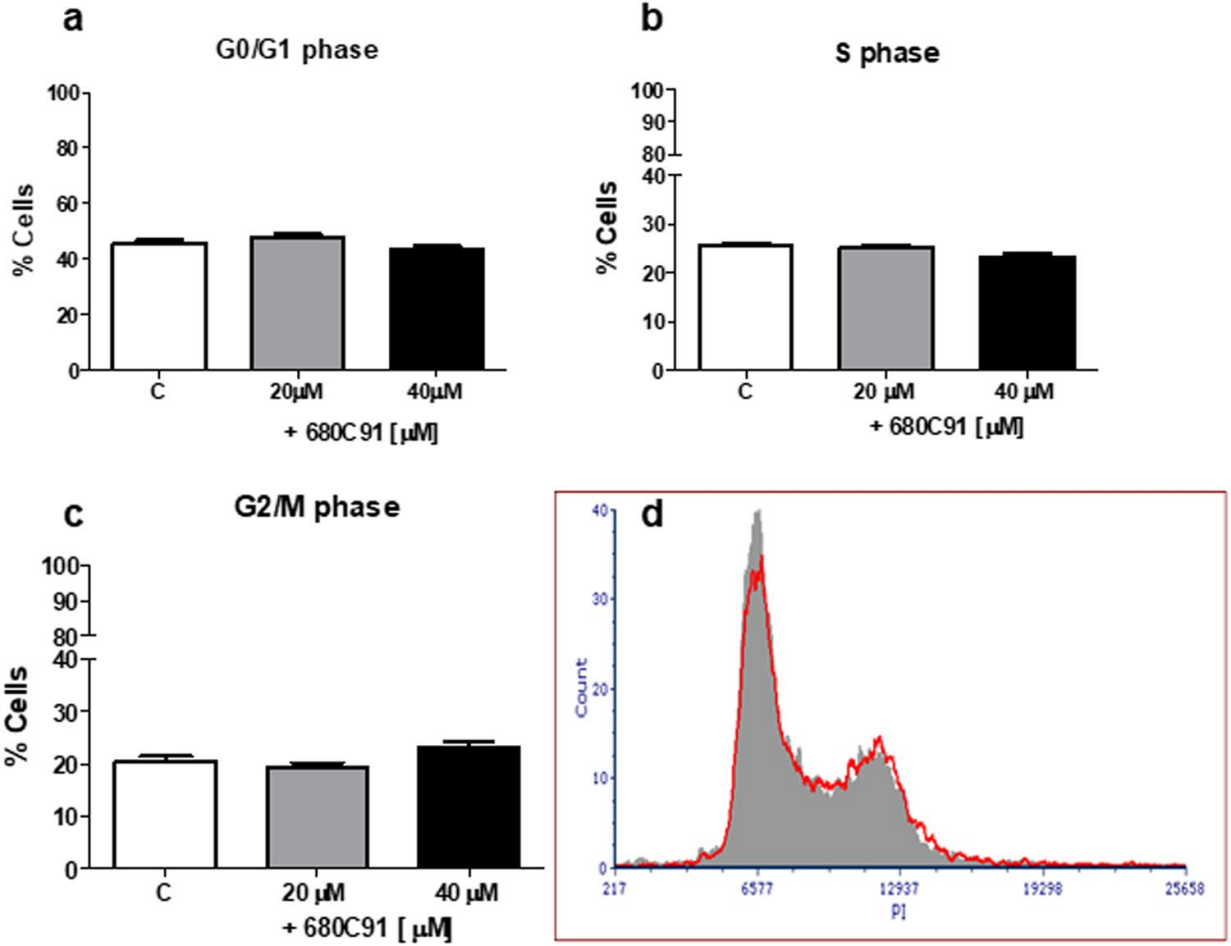
Effect of the TDO inhibitor 680C91 (20 and 40 µM) on HCT-8 cell cycle. Mean ± SEM. **a** G0/G1 phase; **b** S phase; **c** G2/M phase; **d** close to bar graphs there is a representative histogram of control cells (grey line) and 680C91-treated cells (red line)

TDO inhibitor significantly impaired the cell cycle of HUVEC cells (Fig. 4), since a decrease of cell percentage in the G2/M phase was observed, at both concentrations (− 68%). Interestingly, we also observed a decrease in the S phase and an increase of the percentage of cells in the G0/G1 phase. Conversely, 680C91 did not affect HCT8 cell cycle, as shown in Fig. 5.

### Effect of 680C91 on cell apoptosis

**Fig. 6:**
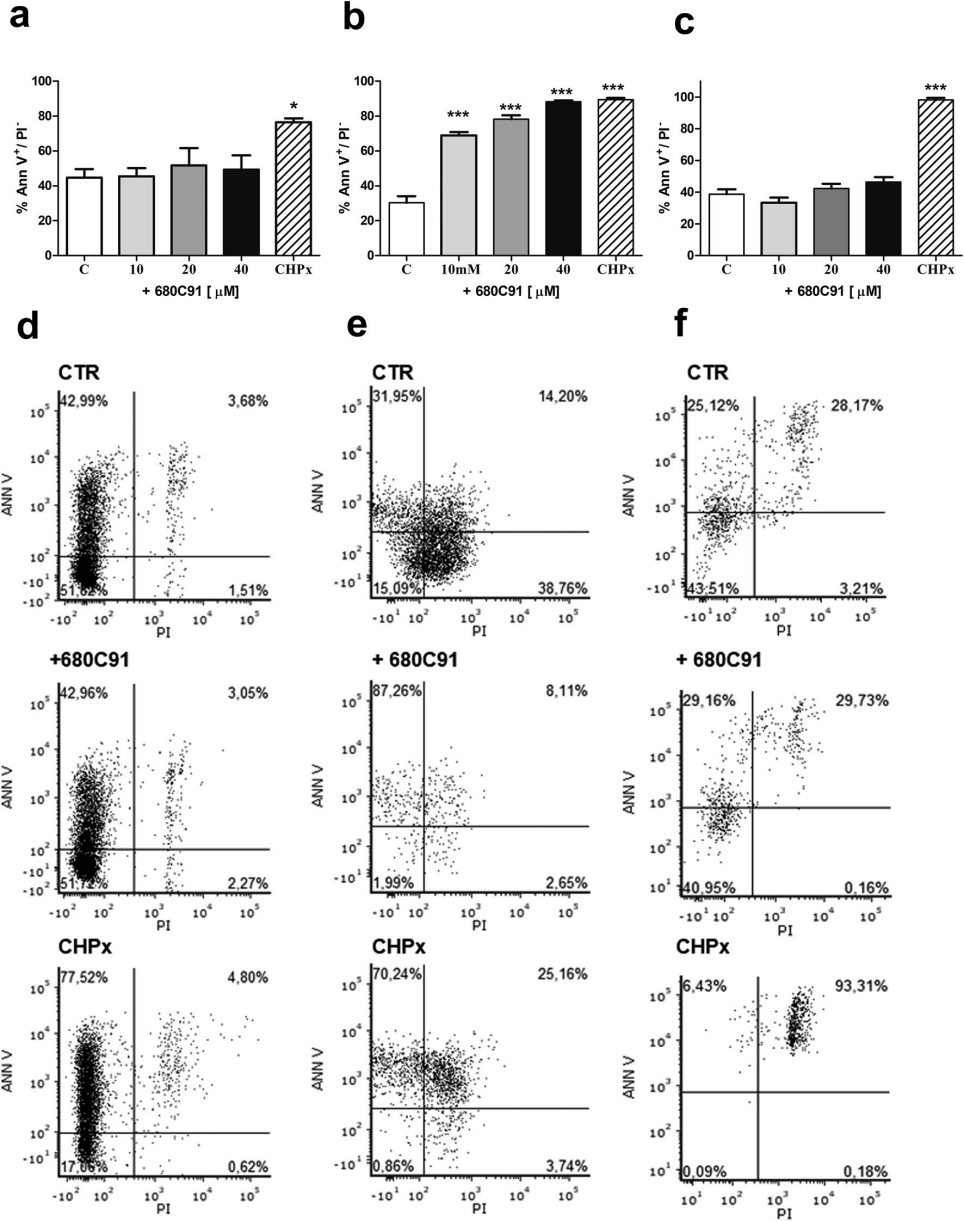
Effect of the TDO inhibitor 680C91 on SK-Mel-28 (**a**, **d**) HCT-8 (**b**, **e**) and HUVEC (**c**, **f**) apoptosis. **a**–**c** Bar chart showing increased proportion of early apoptotic cells. Mean ± SEM, *P < 0.005, ***P < 0.001 vs control unstimulated cells (**c**). Positive control: CHPx, 500 μM **d–f**. Representative figures showing population of viable (annexin V^−^ PI^−^), early apoptotic (annexin V^+^ PI^−^), late apoptotic (annexin V^+^ PI^+^) and necrotic (annexin V^−^ PI^+^) cells

To investigate whether TDO inhibition exerts its antiproliferative activity via induction of apoptosis, cells were treated with varied doses of 680C91 and subjected to FITC-Annexin V/PI staining and analysis by a flow cytometer for measuring early apoptosis (Fig. 6), late apoptosis and cell death (Table 1). Inhibition of TDO did not stimulate SK-Mel-28 (Fig. 6a, d) nor HUVEC (Fig. 6c, f) apoptosis (early and late), nor cell death at any concentration tested (Table 1). Conversely, 680C91 significantly stimulated HCT-8 early apoptosis in a concentration dependent manner (Fig. 6b, e), with a maximal effect induced by 40 µM which induced apoptosis in 88.17 ± 0.88% of cells, comparable to the positive control (CHPx).

**Table 1:** Analysis of cell death and late apoptosis in response to TDO inhibition

	SK-Mel-28		HCT-8		HUVEC	
	C	+ 680C91	C	+ 680C91	C	+ 680C91
Late apoptosis Ann^+^ PI^+^	6.65 ± 0.8	8.76 ± 2.56	4.14 ± 0.13	6.57 ± 0.43	28.9 ± 0.93	28.9 ± 1.62
Dead Ann^−^ PI^+^	0.41 ± 0.17	0.72 ± 0.36	0.06 ± 0.01	0.13 ± 0.02	1.93 ± 0.65	0.16 ± 0.05

Apoptosis was assessed for untreated and 680C91-treated cells (40 µM) by means of flow cytometry. Percentage of late apoptosis was calculated for cells Annexin V^+^/ PI^+^; cells dead as Annexin V^−^/PI^+^. C: control unstimulated cells. Mean ± SEM, *n* = 3

## DISCUSSION

IDO and TDO are the main enzymes within the main pathway of trp degradation, the kynurenine pathway (KP), which produces biologically active metabolites closely correlated with several diseases including inflammation diseases, diabetes, mental disorders, and cancer (Ye et al. 2019). It is known that trp catabolism mediated by IDO is an important mechanism of peripheral immune tolerance contributing to the tumoral immune escape, so that IDO inhibition is an active area of drug development (Pilotte et al. 2012). Despite promising results of IDO1 inhibitors in combination with checkpoint inhibitors, their benefits in melanoma patients have not been completely demonstrated (Kozlova and Frédérick 2019; Long et al. 2019). The latest phase III trial ECHO-301 have reported that the combination of epacadostat (IDO1 selective inhibitor) and PD-1 inhibitor pembrolizumab did not provide any additional survival benefit compared to pembrolizumab alone in advanced melanoma patients (Long et al. 2019). These findings indicate that IDO1-specific inhibitors cannot completely block the production of immunosuppressive trp catabolites involved in the immune escape, suggesting that TDO comes into play (Muller et al. 2019; Sari et al. 2019).

Although it has been shown that TDO has immunomodulatory functions in promoting tumour immune resistance (Pantouris and Mowat 2014), however the role of TDO in human melanoma is still under investigation. This manuscript shows, for the first time, that the human melanoma cell line SK-Mel-28 expresses TDO both on the mRNA (TDO2) and protein levels. Its functional characterization was compared with the one of a human ileocecal adenocarcinoma cell line (HCT-8) and with the one of human venular endothelial cells (HUVEC). IDO and TDO have an important physiological immune suppression role during pregnancy to avoid foetus rejection. HUVECs have been reported to express IDO1 (Beutelspacher et al. 2006) but no information is available for TDO. IDO1 has also been shown to be expressed in colorectal cancer (CRC) cell lines, and its expression at the tumour invasion front is involved in CRC progression (Ferdinande et al. 2012). No information is reported for TDO expression and function in HCT-8 cells. However, in a recent study, it has been found that TDO enzyme was elevated in 40% of the samples from colon cancer patients, while TDO2 mRNA was reduced in few colon cancer cells lines. Surprisingly, human colon cancer samples displayed a more robust up-regulation in the expression of trp transporters and enzymes in the kyn pathway, compared to the expression of the same samples evaluated in vitro, suggesting that this pathway is more active in vivo (Venkateswaran et al. 2019). These findings could explain in our results the lesser amount of TDO2 mRNA expressed by HCT-8 cells, compared to the one of Sk-Mel-28 cells. However, the selective TDO inhibitor used, 680C91, confirmed that TDO is functional in HCT-8 cells.

TDO2 was highly expressed in the human melanoma cell line SK-Mel-28, which has never been reported until now. We then investigated its possible physiological role using a selective TDO inhibitor 680C91 (Salter and Pogson 1985). Firstly, the block of TDO was assessed on cell proliferation in optimal growth conditions (complete medium). The inhibition of TDO inhibited significantly and dose-dependently the cell duplication with a maximal activity obtained with 40 μM. 680C91 was previously reported to reduce triple-negative breast cancer (TNBC) proliferation, migration, and invasion (D’Amato et al. 2015). Elevated TDO expression was indeed associated with an increased disease grade, an estrogenic receptor-negative status, and finally a shorter overall survival. The Authors also demonstrated that pharmacologic inhibition or genetic knockdown of TDO increased sensitivity of TNBC cells to anoikis in a forced suspension culture.

To investigate the antiproliferative effect of 680C91 more in detail, we assessed TDO inhibition both on cell cycle and cell apoptosis. Interestingly, antiproliferative mechanisms of 680C91 were different between cancer cell lines and HUVECs. Inhibition of SK-Mel-28 and HUVEC cell proliferation was due to cell cycle arrest in G2/M phase (Figs. 3 and 4) within 24 h. Conversely, 680C91 did not affect cell cycle but promoted early apoptosis of HCT-8 cells. No cell death was observed, at least within 24 h incubation with TDO inhibitor (Table 1). Therefore, inhibition of TDO has a dichotomous effect: pro-apoptotic for HCT-8 cells and anti-proliferative for the endothelium and SK-Mel-28 cells.

There are some informations on the pro-angiogenic role of IDO in experimental models both in vivo and in vitro (Nonaka et al. 2011; Pan et al. 2017; Zhang et al. 2019). Furthermore, IDO1 has been suggested as a target in lung cancer since its expression is associated with the microvessel density (Pan et al. 2017). The inhibition of endothelial proliferation suggests that TDO may be involved in angiogenesis. Recently, it has been discovered that some type of cancers (such as hepatocarcinoma, glioblastoma, bladder carcinoma) contained foci of non-tumoral TDO-expressing cells, which were identified as pericytes, that were found in high-grade tumours close to necrotic or hemorrhagic areas, characterized by neoangiogenesis (Hoffmann et al. 2020). Although these important observations on IDO1, no data are available on TDO and angiogenesis and on its cooperation with IDO1 on melanoma progression.

These data add new insights on TDO and cancer biology since it may directly modulate cancer cell function and cancer microenvironment rather than immune suppression. Current researches on KP are actually focused on its immunological function with very few studies on its role in the tumour microenvironment. Many studies have focused on IDO, while TDO data are almost completely missing. As previously reported by Optiz (2011), TDO/KP could be involved in cancer biology particularly when IDO does not account for the constitutive trp catabolism, such as in glioma brain tumours. In this study, it was demonstrated that TDO-derived kyn suppresses antitumor immune responses and stimulates tumour-cell survival and migration through the AHR in an autocrine/paracrine fashion (Opitz et al. 2011).

It is mandatory that further investigations on the role of TDO in tumour biology and in particular in the progression of melanoma are needed to find new therapeutic strategies. Recently, an interesting study on the metabolomic identification of human serum markers for advanced melanomas, pointed out that trp levels in metastatic patients were significantly lower, at least 0.2 times than healthy human control samples. These data confirmed the importance of cellular metabolic processes as a hallmark of malignant transformation and or tumour progression (Bayci et al. 2018).
